# Untargeted Metabolomics Reveals Antidepressant Effects in a Marine Photosynthetic Organism: The Diatom *Phaeodactylum tricornutum* as a Case Study

**DOI:** 10.3390/biology11121770

**Published:** 2022-12-06

**Authors:** Bernardo Duarte, Eduardo Feijão, Ricardo Cruz de Carvalho, Irina A. Duarte, Ana Patrícia Marques, Marisa Maia, Jasmine Hertzog, Ana Rita Matos, Maria Teresa Cabrita, Isabel Caçador, Andreia Figueiredo, Marta Sousa Silva, Carlos Cordeiro, Vanessa F. Fonseca

**Affiliations:** 1 MARE—Marine and Environmental Sciences Centre & ARNET—Aquatic Research Network Associated Laboratory, Faculdade de Ciências da Universidade de Lisboa, Campo Grande, 1749-016 Lisbon, Portugal; 2 Departamento de Biologia Vegetal, Faculdade de Ciências da Universidade de Lisboa, Campo Grande, 1749-016 Lisbon, Portugal; 3 cE3c, Centre for Ecology, Evolution and Environmental Changes, Faculdade de Ciências da Universidade de Lisboa, Campo Grande, Edifício C2, Piso 5, 1749-016 Lisbon, Portugal; 4 Laboratório de FT-ICR e Espectrometria de Massa Estrutural, Departamento de Química e Bioquímica, Faculdade de Ciências da Universidade de Lisboa, Campo Grande, 1749-016 Lisbon, Portugal; 5 Université de Lorraine, LCP-A2MC, F-57000 Metz, France; 6 BioISI—Biosystems and Integrative Sciences Institute, Plant Functional Genomics Group, Departamento de Biologia Vegetal, Faculdade de Ciências da Universidade de Lisboa, Campo Grande, 1749-016 Lisbon, Portugal; 7 Centro de Estudos Geográficos (CEG), Instituto de Geografia e Ordenamento do Território (IGOT), Universidade de Lisboa, Rua Branca Edmée Marques, 1600-276 Lisbon, Portugal; 8 Associated Laboratory Terra, 1349-017 Lisbon, Portugal; 9 Departamento de Biologia Animal, Faculdade de Ciências da Universidade de Lisboa, Campo Grande, 1749-016 Lisbon, Portugal

**Keywords:** fluoxetine, metabolomics, ecotoxicology, marine diatoms

## Abstract

**Simple Summary:**

Pharmaceutical compounds, designed to act specifically in human or animal organisms, may have unknown and undesirable effects on non-target organisms in the environment. Untargeted metabolomic analysis uncovered several common effects of fluoxetine in diatoms and mammal cells, specifically in shared metabolic pathways, revealing a common mode of action, including serotonin re-uptake, which is the molecular target of this antidepressant. Moreover, FT-ICR-based metabolomic profiling was shown to be a powerful tool for the identification of potential biomarkers of exposure in diatom cells, highlighting its value in the ecotoxicology of marine organisms.

**Abstract:**

The increased use of antidepressants, along with their increased occurrence in aquatic environments, is of concern for marine organisms. Although these pharmaceutical compounds have been shown to negatively affect marine diatoms, their mode of action in these non-target, single-cell phototrophic organisms is yet unknown. Using a Fourier-transform ion cyclotron-resonance mass spectrometer (FT-ICR-MS) we evaluated the effects of fluoxetine in the metabolomics of the model diatom *Phaeodactylum tricornutum*, as well as the potential use of the identified metabolites as exposure biomarkers. Diatom growth was severely impaired after fluoxetine exposure, particularly in the highest dose tested, along with a down-regulation of photosynthetic and carbohydrate metabolisms. Notably, several mechanisms that are normally down-regulated by fluoxetine in mammal organisms were also down-regulated in diatoms (e.g., glycerolipid metabolism, phosphatidylinositol signalling pathway, vitamin metabolism, terpenoid backbone biosynthesis and serotonin remobilization metabolism). Additionally, the present work also identified a set of potential biomarkers of fluoxetine exposure that were up-regulated with increasing fluoxetine exposure concentration and are of high metabolic significance following the disclosed mode of action, reinforcing the use of metabolomics approaches in ecotoxicology.

## 1. Introduction

The search for an increased life expectancy, better disease prevention and general improvement of human health quality and well-being has led to the progress of medical science and the continuous formulation of more efficient pharmaceutical drugs aiming to reduce the impact of many pathologies [1]. Consequently, we continue to see a substantial increase in the global pharmaceutical market in terms of both the number of newly synthesized compounds and sales, with this trend expected to continue associated with increased access in developing countries and an ageing population in developed countries [2]. This enhanced usage has led to an escalation of pharmaceutical residues found in the environment [1], namely in marine ecosystems [3,4] and even in remote locations such as Antarctica [5]. Although these compounds are typically designed to target specific human receptors, molecules and metabolic pathways at very low concentrations, pharmaceutically active ingredients have the potential to interfere with biochemical and physiological processes in non-target species, with virtually unknown long-term effects in marine ecosystems [1]. These compounds have been detected in several groups of marine organisms, from vertebrates to invertebrates as well as in phytoplankton [3,5,6], and several studies showed that even on non-target organisms, pharmaceutical residues produce negative metabolic effects under environmentally relevant concentrations [6,7,8,9,10,11].

Antidepressants are commonly prescribed for depressive disorders and have elevated risk levels for aquatic organisms [9,10,12,13]. The consumption of these classes of pharmaceutics is high in developed countries and has been amplified by the COVID-19 pandemic [14]. Fluoxetine is a selective serotonin re-uptake inhibitor (SSRI) [15], highly resistant to hydrolysis and photolysis, and therefore persistent in the environment [16]. Given its extensive use, it is not surprising that fluoxetine has been detected in aquatic ecosystems worldwide in concentrations up to 40 µg/L [4,17,18,19]. This compound acts by strongly and selectively inhibiting a transporter enzyme for serotonin re-uptake at the presynaptic membrane, causing an increase in serotonin (5-hydroxytryptamine) concentrations in postsynaptic receptor sites [20]. Previous studies showed that exposure to the antidepressant fluoxetine resulted in decreased feeding and oxidative stress in fish species [9,21,22], and decreased primary productivity in marine diatoms [10]. Although taxa other than animals lack a nervous system, previous research has shown that fluoxetine has severe impacts at different biochemical levels on marine phototrophic organisms, such as diatoms [10]. This is of great concern as diatoms usually account for a large proportion of the primary producers at the basis of marine trophic webs. Therefore, it is fundamental to understand the impacts of fluoxetine at the metabolic level in non-target organisms, such as the model diatom *Phaeodactylum tricornutum*, to also better understand the metabolic pathways governing the toxicological effects observed in prototrophic organisms [10,23,24,25].

The application of high-resolution and high-mass accuracy instruments, based on Fourier-transform technology, is an efficient solution to achieve maximum metabolome coverage, with a sensitivity typically pg level [26,27]. Fourier-transform ion cyclotron-resonance mass spectrometer (FT-ICR-MS) is the highest performance equipment within this category, providing ultra-high-mass accuracy (below 1 ppb) and the highest mass resolution (more than 1,000,000), being able to use direct infusion coupled to ultra-high-resolution mass spectrometry, enabling metabolite analysis in a high-throughput and rapid, largely unbiased way, of complex metabolite samples, while simultaneously eliminating the time-consuming separation by liquid chromatography (LC) [26,27].

In the present work, we aimed to disentangle the metabolic effects of the human antidepressant fluoxetine exposure on a marine model diatom (*P. tricornutum*) using state-of-the-art untargeted FT-ICR-MS metabolomics and evaluate its potential to identify key metabolites as exposure biomarkers.

## 2. Materials and Methods

### 2.1. Diatom Growth and Fluoxetine Exposure

For the present work, we used a monoclonal *P. tricornutum* Bohlin (Bacillariophyceae) (strain IO 108-01, Instituto Português do Mar e da Atmosfera (IPMA)) axenic cell culture kept under asexual reproduction conditions in f/2 medium [28]. The axenic state of the cultures was ensured through periodic visual inspection under the microscope. All manipulations were executed within a laminar flow hood chamber, ensuring standard aseptic conditions. *Phaeodactylum tricornutum* cells (1 mL volume sample) were counted using a Neubauer improved counting chamber, coupled with an Olympus BX50 (Tokyo, Japan) inverted microscope, at 400-times magnification. For exposure purposes, cultures were maintained under constant aeration in a phytoclimatic chamber, at 18 °C, programmed with a 14/10 h day/night photoperiod (RGB 1:1:1, maximum PAR 80 μmol photons m^−2^ s^−1^), a sinusoidal function to mimic sunrise and sunset, and light intensity at noon, set to replicate a natural light environment [29]. Exposure was performed according to the Organisation for Economic Cooperation and Development (OECD) recommendations for algae assays [30], with minor adaptations, and the suggested initial cell density for microalgae cells with comparable dimensions to *P. tricornutum* (initial cell density = 2.7 × 10^5^ cells mL^−1^). Aeration with ambient air was provided as the main carbon source. Due to the fast growth rates of this diatom, the exposure time was reduced to 48 h, to avoid artefacts to the ageing of the cells observed at 72 h, when the cultures are already in the stationary phase [29]. After 48 h acclimation, fluoxetine was added to cell cultures to attain target concentrations (0, 0.2, 20 and 80 μg/L), in a total of 12 experimental units (4 fluoxetine concentrations tested with 3 replicates each). All exposures were performed simultaneously. Concentrations were selected to cover a concentration gradient reflecting current environmental concentrations [31,32,33], as well as a higher concentration to reflect the continued increase in the use of fluoxetine [34,35] and previous physiological results obtained [10]. Exposure occurred for another 48-h period. Growth inhibition was calculated according to the OECD recommendations for algae assays [30]. At the end of the 48 h exposure period, cells were harvested for biochemical analysis by centrifugation at 4000× *g* for 15 min at 4 °C. The resulting pellets were frozen in liquid nitrogen and stored at −80 °C. Three biological replicates for all tested conditions were considered for each analysis and collected from a total of 12 cultivation units.

### 2.2. Metabolite Extraction and Fourier-Transform Ion Cyclotron-Resonance Mass Spectrometer (FT-ICR-MS) Analysis

Sample pellets were extracted with 200 mL of methanol/water (1:1), vortexed for 5 min and cooled on ice. This procedure was repeated five times. After centrifugation, the supernatant was recovered and diluted 100-fold in methanol (10 mL sample in 990 mL methanol). Human leucine enkephalin (YGGFL, Sigma Aldrich, St. Louis, MO, USA) was added at a concentration of 0.5 mg/mL, for internal mass spectrum calibration ([M + H]^+^ = 556.27658 Th). Samples were introduced in the electrospray ionization (ESI) source by direct infusion, using a kdScientific syringe pump at a flow rate of 2 μL/min in a SolariX XR 7-Tesla Fourier-Transform Ion Cyclotron-Resonance Mass Spectrometer (FT-ICR-MS, Bruker Daltonics, Bremen, Germany), equipped with the Paracell, operating in positive (ESI+) ionization mode. For ESI+, 0.1 % (*v*/*v*) formic acid (Sigma Aldrich, MS grade) was added to the sample before analysis. Mass spectra were acquired in absorption mode, 4 MB transient length, 100 transients were accumulated per spectrum in the mass range of 100–1000 *m*/*z*.

### 2.3. Data Analysis

FT-ICR-MS spectra were processed using Compass Data Analysis 5.0 (Bruker Daltonics, Bremen, Germany). Each mass spectrum was further internally calibrated using a list of eight known chemical formulas detected in the form of [M + H]^+^ ions (C_6_H_15_N_4_O_2_-*m*/*z* 175.118952; C_7_H_14_NaO_7_-*m*/*z* 233.063173; C_16_H_32_O_2_Na-*m*/*z* 279.229451; C_18_H_36_O_2_Na-*m*/*z* 307.260751; C_20_H_30_NaO_2_-*m*/*z* 325.213801; C_14_H_30_NaO_8_-*m*/*z* 349.183289; C_15_H_28_NaO_13_-*m*/*z* 439.142212; C_28_H_38_N_5_O_7_ (the internal standard leucine enkephalin)-*m*/*z* 556.276575) for which the mass accuracy values were lower than 200 ppb. Each peak list was exported with a minimum signal-to-noise ratio of 6. Signals related to magnetron and satellite peaks were removed using an algorithm developed by Kanawati et al. [36]. Peak lists were aligned considering a deviation of 0.5 ppm and *m*/*z* values present in more than 50% of the replicate samples were conserved. After replica alignment and filtration, 3362 peaks were used for further statistical analyses.

Data normalization and fold-change determination were performed with MetaboAnalyst version 5.0 (https://www.metaboanalyst.ca/home.xhtml) (accessed on 30 October 2022) [37]. Missing value imputation was made using substitution, considering half of the minimum positive value found within the data. Intensity data were log-transformed and Pareto-scaled before statistical analysis by multivariate methods. Pathway analysis was also performed in MetaboAnalyst version 5.0, using the normalized intensity data.

Retrieved peak lists were submitted to the MassTRIX 3 server (http://masstrix.org, accessed on 30 May 2022) [38] for putative compound identification, using the *P. tricornutum* available database as a baseline with K and Na adducts. A maximum *m*/*z* deviation of 1 ppm was accepted; “KEGG (Kyoto Encyclopaedia of Genes and Genomes)/HMDB (Human Metabolome Database)/LipidMaps without isotopes” was selected for database search. Compounds with valid KEGG identification were submitted to KEGG *P. tricornutum* metabolic pathway mapper (https://www.genome.jp/kegg-bin/show_organism?menu_type=pathway_maps&org=pti) for visualization (accessed on 30 October 2022).

All statistical analyses were performed in R-Studio Version 1.4.1717. Spearman correlation coefficients and statistical significance between the metabolomic traits and the fluoxetine exogenous concentration were computed using the *corrplot* package [39]. Heatmaps and boxplots with probability densities of the data at different values smoothed by a kernel density estimator were computed and plotted using the *ggplot2* package [40]. Non-parametric Kruskal–Wallis tests with Bonferroni correction, for comparisons between the variables and samples exposed to different fluoxetine concentrations, were performed using the *agricolae* package [41]. Venn diagrams used to analyse which compounds appeared exclusively or simultaneously in control versus fluoxetine-exposed samples were performed using InteractiVenn [42]. Partial least-squares discriminant analysis (PLS-DA), variable importance in projection partial least-squares discriminant analysis (VIP-PLS-DA) and Clustered Image Map (CIM) were performed using the *DiscriMiner* package [43].

## 3. Results

### 3.1. Diatom Growth

**Figure 1:**
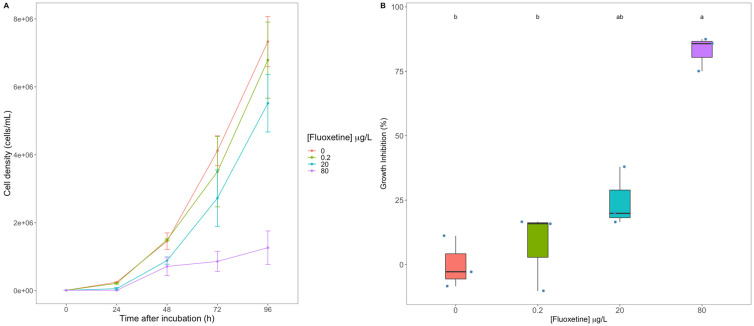
Growth curves ((**A**), average ± standard error) and growth inhibition (**B**) observed in the diatom cultures exposed to 0, 0.2, 20 and 80 μg/L fluoxetine (*n* = 3, different letters denote significant differences at *p* < 0.05).

Diatom growth was strongly affected following culture exposure to fluoxetine, with increasing concentrations resulting in a progressive decrease in cell density, and cultures exposed to 80 μg/L fluoxetine displayed the lowest cell density at the end of the trial (96 h) (Figure 1A). This resulted in significantly higher growth inhibitions, particularly in the cultures exposed to 80 μg/L fluoxetine (Figure 1B). The cultures subjected to the lowest fluoxetine concentrations tested (0.2 and 20 μg/L) did not reveal any significant growth inhibition when compared to the control conditions.

### 3.2. Fluoxetine Impacts on Diatom Metabolisms and Pathways

**Figure 2:**
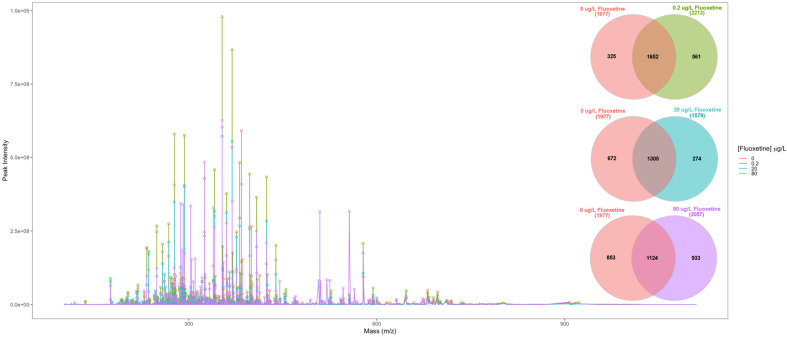
Fourier-transform ion cyclotron-resonance (FT-ICR-MS) aligned averaged mass spectra of the diatom extracts, corresponding to cells exposed to 0, 0.2, 20 and 80 μg/L fluoxetine and Venn diagrams of the distinct number of m/z peaks detected between the control condition (0 μg/L) and the fluoxetine exposure conditions (average, *n* = 3 per treatment).

A first analysis of the FT-ICR-MS spectra from the cells exposed to different fluoxetine concentrations revealed a wide array of changes, both in terms of peak intensity and peak diversity (Figure 2). This is particularly evident in the mass spectra corresponding to the peaks obtained from the cells exposed to the highest fluoxetine dose (80 μg/L), where the lowest number of common compounds were detected between this treatment and the control conditions (0 μg/L). Moreover, the cells exposed to 80 μg/L also showed the highest number of exclusively detected peaks (933), when compared to the control condition.

**Figure 3:**
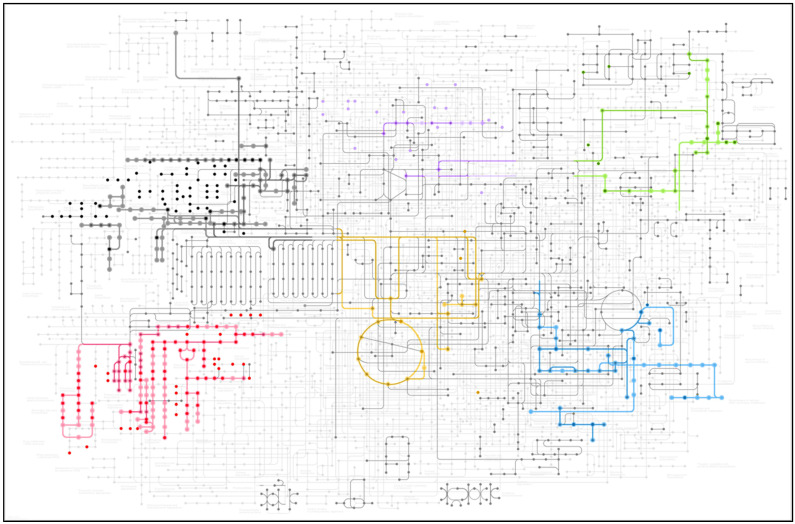
Overview of the KEEG pathways (coloured lines) and putative metabolites (highlighted dots) identified using the MassTRIX, with the Fourier-transform ion cyclotron-resonance aligned mass spectra of the diatom exposed to fluoxetine as a baseline.

A total of 640 putative metabolites that were significantly up- or down-regulated following fluoxetine exposure were identified upon submission of the mass data of all surveyed samples to the MassTRIX platform, and several metabolic pathways were highlighted in the KEGG database (Figure 3). Six major metabolic pathways were highlighted due to the high number of metabolites belonging to these pathways and identified by MassTRIX. These metabolic pathways are mostly linked to the photosynthetic carbon fixation (yellow pathway), glycolysis (blue pathway), vitamin metabolism (green pathway), carbohydrate metabolism (purple pathway), fatty acid and lipid metabolism (black pathway) and steroid metabolism (red pathway).

**Figure 4:**
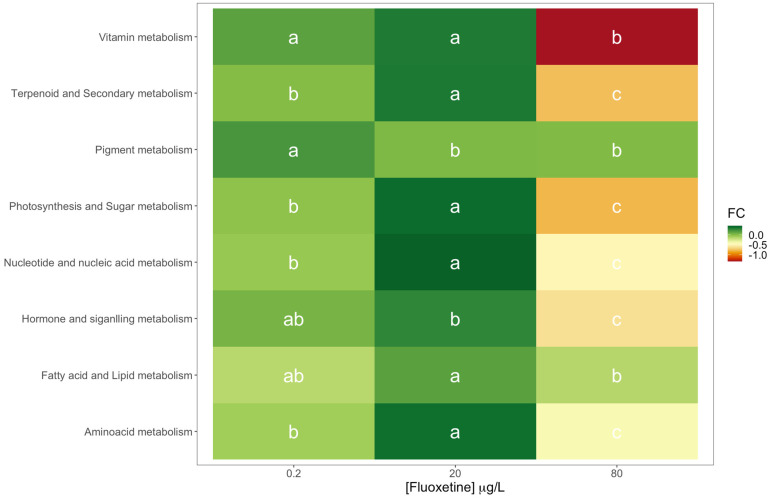
Fold-change heatmap of the different metabolic networks identified in the diatom cells exposed to 0.2, 20 and 80 μg/L fluoxetine (*n* = 3, different letters denote significant differences at *p* < 0.05).

A more detailed analysis of the different diatom metabolic networks affected by fluoxetine exposure indicated that these were differentially affected both in qualitative (up or down-regulated) and in intensity (fold-change) terms. Analysing the results in terms of metabolic networks (Figure 4) fold-change variations towards the control condition (0 μg/L fluoxetine), several differences were evident. Most of the metabolic networks evaluated were significantly down-regulated following fluoxetine exposure to the highest tested concentration (80 μg/L). This down-regulation was more pronounced in the metabolites involved in vitamin metabolism, terpenoid and secondary metabolism, photosynthesis and carbohydrate metabolism, and hormone and signalling metabolisms.

Fatty acid and lipid metabolism was the only up-regulated metabolic network observed, with significantly higher fold-change values observed in the diatom cultures exposed to the highest fluoxetine concentration tested. Maximum fold-change values for most of the metabolic networks (apart from pigment and hormone and signalling metabolisms) were observed in the individuals exposed to intermediate fluoxetine concentrations.

**Figure 5:**
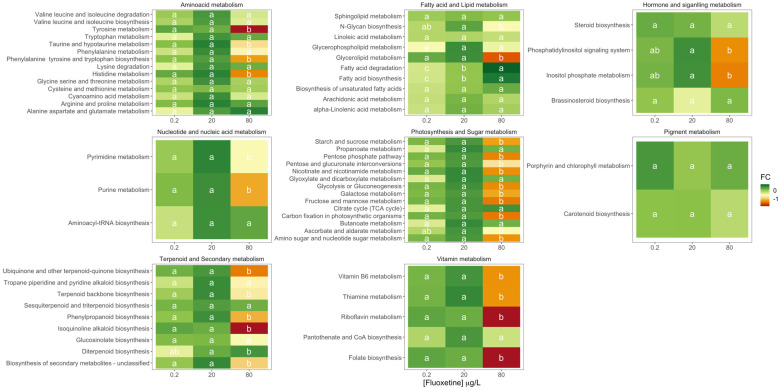
Fold-change heatmap of the different metabolic pathways of each metabolism group identified in the diatom cells exposed to 0.2, 20 and 80 μg/L fluoxetine (*n* = 3, different letters denote significant differences at *p* < 0.05).

Changes in metabolite regulation were also observed within each of the specific metabolic pathways (Figure 5). In the amino acid metabolism, all the significant differences were associated with the highest fluoxetine dose tested, leading to down-regulation of specific pathways such as tyrosine, taurine and hypotaurine metabolisms, phenylalanine, tyrosine and tryptophan biosynthesis and histidine metabolism. Regarding fatty acid and lipid metabolic pathways, fluoxetine exposure led to a significant rise in the fold-change of the metabolites enrolled in fatty acid degradation and biosynthesis, with both 20 and 80 μg/L fluoxetine treatments related to significant increases. Additionally, the highest fluoxetine concentration tested triggered significant reductions in the fold-change variation of the metabolites involved in the N-glycan biosynthesis and glycerolipids metabolic pathways. In the hormone and signalling metabolism, significant fold-change declines were observed in the metabolites intervenient in the phosphatidylinositol signalling system and the inositol phosphate metabolism. The same tendency could be observed for the metabolites involved in pyrimidine and purine pathways of the nucleotide and nucleic acid metabolism. Photosynthesis and sugar metabolism were severely affected by fluoxetine exposure, notably with the highest tested concentration, with only the metabolites involved in the propanoate, glyoxylate and dicarboxylate metabolisms, citrate cycle and butanoate metabolism presenting no variation along with the tested treatments. No significant changes could be observed in the metabolic pathways involved in pigment metabolism. Analysing the terpenoid and secondary metabolism pathways, significant changes in the metabolite profiles upon fluoxetine application were observed. Apart from sesquiterpenoid and triterpenoid biosynthesis and glucosinolate metabolism, all the remaining metabolic pathways showed significant down-regulation in the cells exposed to the highest fluoxetine concentration tested. The same could be observed in the metabolites enrolled in vitamin metabolism, except for the pantothenate and CoA biosynthesis pathways, which exhibited no variation independently of the fluoxetine dose applied.

### 3.3. Fluoxetine Metabolic Biomarker Discovery

**Figure 6:**
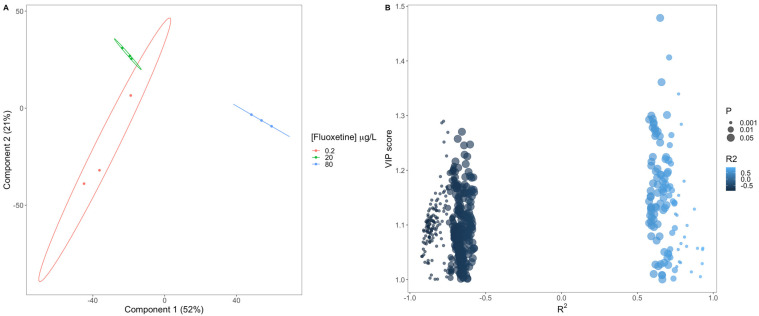
Mass intensity fold-change Variable Importance in Projection Partial Least-Squares Discriminant Analysis (VIP-PLS-DA) biplot (**A**) and a scatterplot of the attained VIP scores and correlation coefficients (R^2^) and significance classes (P) between the calculated fold-change and the fluoxetine exogenous dose applied (**B**), for the detected mass values with VIP score values above 1.

To evaluate the applicability of the assessed metabolite profiles as biomarkers of fluoxetine exposure in *P. tricornutum*, a VIP-PLS-DA analysis was conducted (Figure 6A). The model projection revealed no overlap between the samples based on the mass intensity fold-change. This resulted in 100 % accuracy in the classification of the samples with the produced VIP-PLS-DA model. Considering the variables with higher importance for the PLS-DA projection (VIP scores > 1), its relationship with the exogenous fluoxetine concentration applied to the cultures was evaluated, based on the R^2^ (Figure 6B). From this analysis, it was possible to observe that the majority (79.9%, dark blue circles) of the detected mass values showed fold-changes with decreasing trends along the fluoxetine gradient applied and with a high degree of significance. On the other hand, the detected mass values that presented increasing fold-change values along the fluoxetine concentration gradient (20.1% of the total masses detected) presented the masses with higher VIP scores.

**Figure 7:**
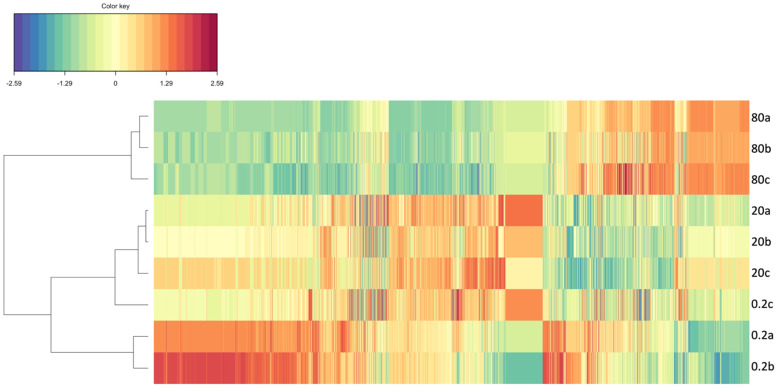
Fold-change Clustered Image Map (CIM) and respective Euclidean Dissimilarity dendrogram based on the metabolite fold-changes of the cells exposed to 0.2, 20 and 80 μg/L fluoxetine and respective replicates (a, b, and c). Each tile represents a metabolite.

The analysis of the fold-change patterns of the detected mass values for each of the surveyed samples (Figure 7), shows a clear separation of the samples into two major groups. The Clustered Image Map dendrogram indicated a clear separation of the samples exposed to the highest fluoxetine concentration (80 μg/L) from the remaining treatments, indicating similar mass fold-change profiles under low and mild fluoxetine exposure. Moreover, a shift in the up and down-regulation pattern was also noticeable when comparing the fold-change profiles of the samples exposed to 80 μg/L fluoxetine with the remaining ones. This was evident in the shift of the down-regulated mass values detected in 0.3 and 20 μg/L to up-regulated values in the samples exposed to 80 μg/L fluoxetine and vice-versa.

**Figure 8:**
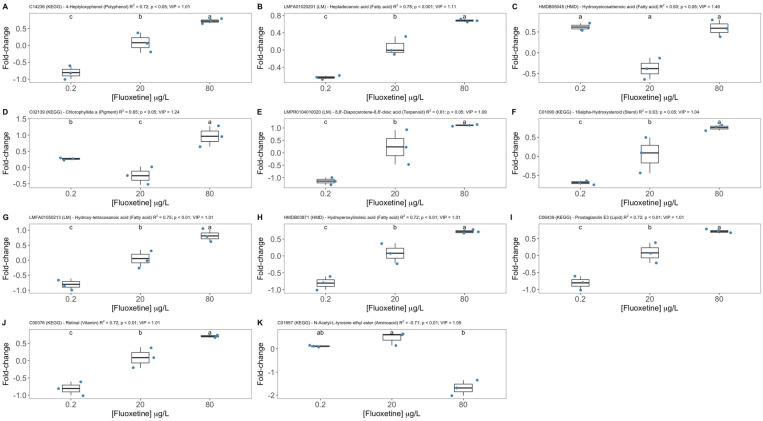
Fold-change of the selected potential biomarkers of fluoxetine exposure in the diatom cells exposed to 0.2, 20 and 80 μg/L fluoxetine (*n* = 3, different letters denote significant differences at *p* < 0.05), database identification code (KEGG—Kyoto Encyclopaedia of Genes and Genomes; HMD—Human Metabolome Database; LM—LipidMaps), fluoxetine-fold-change correlation coefficient (R^2^) and Variable Importance in Projection Partial Least-Squares Discriminant Analysis (VIP-PLS-DA) score.

After identification of the detected mass values using the KEGG library for *P. tricornutum*, the putative metabolites were filtered as potential biomarkers when displaying a VIP score above 1 and a significant correlation between fold-change and the fluoxetine exogenous dose applied to the diatom cultures (Figure 8). This allowed reducing the number of potential biomarkers, due to both the positive identification and the statistical requirements defined. Most of the putative metabolites selected through this procedure belonged to lipid, fatty acid and secondary compounds categories (polyphenols and terpenoids). The metabolites identified as 4-Heptyloxyphenol, Heptadecanoic acid, 8,8′-Diapocarotene-8,8′-dioic acid, 16alpha-Hydroxysteroid, Hydroxy-tetracosanoic acid, Prostaglandin E3 and Retinal displayed a dose-response relationship with statistically significant increments in their fold-change along with the fluoxetine exogenous concentration (Figure 8A,B,E–J, respectively). Other components showed different relationships with fluoxetine, whereby the intermediate 20 μg/L fluoxetine (Figure 8C,D, Chlorophyllide a and N-acetyl-L-tyrosine ethyl ester) or the maximum 80 μg/L fluoxetine concentrations had the minimum response (Figure 8K, Hydroxy-eicosatrienoic acid).

**Figure 9:**
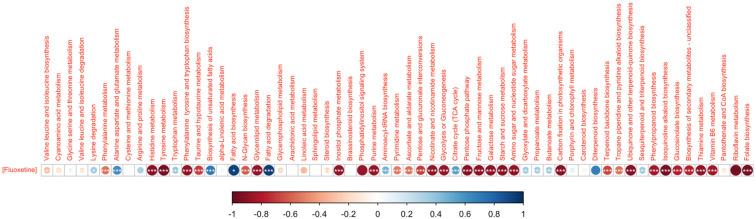
Spearman correlogram between the evaluated metabolic pathways and the exogenous fluoxetine concentration applied to the diatom cultures (asterisks denote significant correlations at * *p* < 0.05, ** *p* < 0.01 and *** *p* < 0.001).

Regarding the potential use of the metabolic networks (a set of metabolites belonging to the same pathway) analysed as biomarkers of fluoxetine exposure, some correlations were observed between several pathways and the exogenous fluoxetine dose applied (Figure 9). In fact, most of the surveyed metabolic pathways showed significant inverse correlations with the exogenous fluoxetine dose applied. Contrastingly, lysine degradation, alanine, aspartate and glutamate and tryptophan metabolisms, biosynthesis of fatty acids (total and unsaturated) as well as their degradation metabolism, aminoacyl-tRNA biosynthesis, citrate cycle, glyoxylate and dicarboxylate, propanoate and butanoate metabolisms, and sesquiterpenoid and triterpenoid biosynthesis showed significant direct correlations with the fluoxetine concentration applied to the diatom cultures.

## 4. Discussion

Although fluoxetine is a neuroactive drug designed to target the human synaptic serotonin reabsorption metabolism, its effects in non-target organisms have been reported in algae, fish and mammals including humans [9,10,11,21,22,44,45,46]. Moreover, the results from these studies showed significant side effects of fluoxetine exposure, which go beyond its primary target in the synaptic serotonin reabsorption, including affecting organisms lacking central nervous systems such as diatoms [10,45,46]. Not only because fluoxetine is one of the most commonly prescribed antidepressants but also because of its prevalence in marine systems and organisms [3,4,16,47], it is key that we understand how fluoxetine affects non-target organisms and what are the metabolic implications and effects behind the observed toxicity.

Diatom growth was severely impaired with fluoxetine exposure, especially in the cells exposed to 80 μg/L dose. This was previously associated with significant decreases in the diatom primary photochemistry metabolism [10,24,46], and our results suggest is supported by the down-regulation of the photosynthesis and carbohydrate metabolism observed here upon 80 μg/L fluoxetine application. In fact, our results suggest the only photosynthesis and carbohydrate pathways that were not affected by fluoxetine exposure were the citrate cycle, propanoate, butanoate and glyoxylate metabolic pathways, indicating severe effects on the overall carbon fixation mechanisms and thus impairing biomass production. It was found in the previous work that fluoxetine regulates glucose metabolism and inhibits the phosphatidylinositol 3-kinase/protein kinase B (PI3K-AKT) signalling pathway [48]. These results are in line with the observed significant down-regulation of carbohydrate metabolism suggested by our results, namely glycolysis and glycogenesis, but also a significant down-regulation of the inositol phosphate and phosphatidylinositol signalling pathways following the exposure to high exogenous fluoxetine concentrations. Fluoxetine is known to affect lipid metabolism, namely impairing the glycerolipids metabolic pathway due to PI3K-AKT pathway disruption [48]. Our results suggest that fluoxetine exposure in *P. tricornutum* also down-regulated the glycerolipids metabolic pathway at high fluoxetine concentrations. Overall, fluoxetine exposure resulted in similar effects in mice and diatoms due to a down-regulation of common metabolic pathways and a cascade of events triggered by the down-regulation of the phosphatidylinositol signalling pathway [48].

The results presented also suggest that fatty acid biosynthesis was also significantly up-regulated along the fluoxetine gradient applied to the diatom cultures. Fatty acid synthase (FAS) and acetyl-CoA carboxylase (ACC) are important enzymes that regulate the process of fatty acid synthesis [49]. Previous studies [50] have shown that in mice fluoxetine induced an increase in the expression of ACC and FAS, thus up-regulating the fatty acid biosynthesis. Again, this appears to be a common feature, as it was also evident in diatoms. Simultaneously, our results point to an up-regulation of the diatom fatty acid degradation pathway under fluoxetine exposure. This antidepressant is known to affect brain phospholipase A2 increasing its activity and thus promoting fatty acid degradation by increased phospholipid fatty acid cleavage [51]. Interestingly, in fluoxetine-exposed diatom cells, the increased amounts of the oxylipins (fatty acid oxidation products) hydroxyperoxylinoleic acid, hydroxytetranoic acid, and prostaglandins, also require the removal of polyunsaturated fatty acids from glycerolipids by a PLA-type enzyme [51].

Pathways involved in vitamin metabolism were also severely impaired. Fluoxetine has been reported to significantly decrease vitamin B6 and riboflavin metabolisms, due to its intervention as an essential cofactor in tryptophan metabolism and a key methyl donor in homocysteine conversion [52]. Our results suggest that in fluoxetine-exposed diatom cells, vitamin B6 and riboflavin metabolisms were severely down-regulated, although neither tryptophan nor cysteine amino acid metabolic pathways were affected by fluoxetine exposure. This can be indicative of a common effect on the depletion of vitamin B6 between mammals and diatoms, although linked to different metabolic processes. Folic acid is also known to be depleted with fluoxetine application due to its relevant role in the synthesis of tetrahydrobiopterin, which is the cofactor for the hydroxylation of phenylalanine and tryptophan [53]. The present results also suggest that in diatoms exposed to 80 μg/L fluoxetine, a significant depletion of the folate biosynthesis was evident which would interfere directly with phenylalanine and tryptophan metabolisms [53]. Our results suggest that thiamine metabolism was another pathway that was severely down-regulated by fluoxetine exposure. This vitamin acts as an essential cofactor in carbohydrate metabolism [54] and therefore, its down-regulation could act as a supplemental down-regulatory mechanism of the abovementioned carbohydrate metabolism. The results presented here also suggest that Purine and Pyrimidine metabolisms were also significantly down-regulated by fluoxetine exposure. This down-regulation was already observed in juvenile rhesus monkeys [55]; however, the mechanism of how fluoxetine affects Purine and Pyrimidine metabolisms is still unclear, with prior works suggesting a direct effect of fluoxetine in the purinergic P2Y1 and P2Y12 receptors signalling pathways [56].

The present results also suggest that fluoxetine-exposed diatom cells showed down-regulation of several terpenoid and secondary metabolic pathways with fluoxetine exposure. The ubiquinone (also known as Coenzyme Q10) pathway was significantly down-regulated in *P. tricornutum* cells exposed to 80 μg/L fluoxetine. Agreeing with our results, Fluoxetine administration in rats also led to a decrease in ubiquinone levels as this molecule is a component of the mitochondrial electron transport chain and has well-documented antioxidant properties [57]. Our results suggest that the terpenoid backbone biosynthesis pathway was also down-regulated in the diatom cells exposed to 80 μg/L fluoxetine. A previous metabolomics study analysing urine samples from rats exposed to fluoxetine showed that this compound significantly affected terpenoid backbone biosynthesis [58]. These authors state that terpenoid backbone biosynthesis is associated with energy metabolism, and the disturbances in the energy metabolism (here reported as the abovementioned down-regulations of the photosynthesis and sugar metabolism), would have direct effects on terpenoid backbone biosynthesis [58]. Phenylpropanoid biosynthetic enzyme is actively involved in serotonin methylation to hydroxytryptamine, a precursor of 5-methoxy N-acetyl tryptamine (melatonin), with diverse physiological roles, including growth, reproduction, photoperiodic response, antioxidant and defence mechanisms in plants [59]. The direct impact of fluoxetine on serotonin remobilization has therefore significant impacts on the phenylpropanoid biosynthesis pathway. This is in line with our results, where the phenylpropanoid biosynthesis pathway appears to be significantly down-regulated at high fluoxetine concentrations, indicating that although fluoxetine is designed to inhibit serotonin re-uptake in the nervous system synaptic spaces, the action of this antidepressant is also observable in algae cells in a similar way.

Beyond the undeniable value of state-of-the-art untargeted FT-ICR-MS metabolomics to unravel the metabolic mechanisms behind fluoxetine toxicity in non-target organisms, such as marine diatoms, this technique also provides large datasets that can be used for fluoxetine biomarker discovery. In ecotoxicological terms and considering the diatom *P. tricornutum* as a model species for ecotoxicology, biomarker discovery approaches that can simultaneously provide insights on the mode of action of any potentially toxic compound and be a source of potential biomarkers, are key for future environmental risk assessments. In the past, several biomarkers were developed for this diatom species, focusing specifically on targeted metabolism [23,24,25], thus an untargeted approach would be of added value to provide a more comprehensive evaluation of the whole diatom metabolism. This metabolomic approach to ecotoxicological assays is very recent but promising for marine ecotoxicology [60]. The metabolomic profile of the diatom cells exposed to different fluoxetine concentrations generated very distinct clusters of samples. The VIP-PLS-DA analysis of the diatom metabolome data generated a model with 100% accuracy in classifying the samples according to the fluoxetine exposure dose, using just the fold-change data as descriptors. Crossing the data provided from the VIP scores of the identified compounds with their dose-response relationships and statistical significance, allowed us to filter the number of compounds to key metabolites which can be potential biomarkers of fluoxetine exposure in marine diatoms. Most of the identified metabolites showed an up-regulation due to fluoxetine exposure. Among these, metabolites identified as potential biomarkers comprised five lipid and fatty acid metabolites. This is in line with our previous findings where it was shown that some of the most relevant effects of fluoxetine exposure in *P. tricornutum* occurred at the lipid and fatty acid metabolism [10], with a proposed fatty acid-based integrative index developed for improved ecotoxicological assessments in fluoxetine and other emerging contaminants [23]. Another interesting biomarker identified by this untargeted FT-ICR-MS metabolomic approach was chlorophyll *a* precursor, chlorophyllide *a* [61]. In previous works [10] fluoxetine significantly reduced the chlorophyll *a* content of diatom cells exposed to fluoxetine, and therefore the increase in its precursor can be perceived as counteractive feedback activated in diatom cells to thwart this depletion, which led to a significant reduction of the cells light-harvesting ability and consequently photosynthetic activity [10]. Another interesting biomarker highlighted is retinal, commonly known as vitamin A, a recognized antioxidant molecule. Diatom cells exposed to high fluoxetine concentrations have revealed a high degree of oxidative stress with the activation of several enzymatic antioxidant mechanisms [10,25]. Thus, the up-regulation of vitamin A can be an additional antioxidant mechanism activated by the fluoxetine-exposed cells. Fluoxetine treatment is also known to increase gene expression of prostaglandin biosynthetic enzymes involved in fatty acid mobilization and accumulation [62]. In our results, Prostaglandin E3 showed potential as a biomarker of exposure to this antidepressant with an increasing dose along the fluoxetine gradient applied. Although this is a typical animal metabolite, isoprostanoids such as prostaglandin are produced non-enzymatically in *P. tricornutum* upon oxidative stress challenge [63]. N-acetyl tyrosine ethyl ester was the only biomarker highlighted by the selection procedure showing a decreasing trend along the fluoxetine concentration gradient tested. The down-regulation of this metabolite has been associated with nitrogen reabsorption mechanisms in stress conditions in plants [64], and thus, can be attributed to the diatom cells’ nitrogen needs, e.g., for de-novo chlorophyll synthesis, which is one of the main demands of nitrogen in photosynthetic organisms. In addition to individual metabolite biomarkers, it was also possible to identify metabolic pathways as promising candidates for functional biomarker sets for fluoxetine exposure in marine diatom cells. Most of the surveyed metabolic pathways showed significant inverse correlations with the exogenous fluoxetine dose applied. However, pathways such as lysine degradation, alanine, aspartate, glutamate and tryptophan metabolisms, biosynthesis of fatty acids (total and unsaturated), as well as their degradation metabolism, aminoacyl-tRNA biosynthesis, citrate cycle, glyoxylate, dicarboxylate, propanoate and butanoate metabolisms, and sesquiterpenoid and triterpenoid biosynthesis, were directly correlated with the fluoxetine dose applied, and are in line with the abovementioned metabolic effects of fluoxetine. Thus, these pathways can be used as potential functional biomarkers of fluoxetine exposure.

Although the most significant and evident effects were detected in the cells exposed to the highest fluoxetine concentration tested (80 μg/L), above the present day average maximum concentration detected in aquatic systems (40 μg/L), it should be notice that for several metabolites and metabolic pathways the effects were already felt at exposure doses bellow environmentally relevant concentrations (20 μg/L), indicating that even under lower doses than the maximum tested the presence of this antidepressant in aquatic mediums can be of concern and affect negatively the metabolism of marine diatoms. Moreover, and considering the increasing trend in the use of antidepressants, present day concentrations are expected to be surpassed in the coming years, leading to exacerbated effects in marine primary producers, such as the ones here demonstrated for this model marine diatom.

## 5. Conclusions

Although fluoxetine is designed to target specific synaptic serotonin re-uptake mechanisms, its effects on other metabolic pathways are generally well recognized. Diatoms are far from being the target organisms of this antidepressant, nevertheless, these photosynthetic organisms share several common metabolic pathways with mammal species and are affected by the presence of fluoxetine in the environment. The untargeted metabolomics analysis revealed several effects of fluoxetine in diatoms via shared metabolic pathways with mammal cells, thus demonstrating a common mode of action, including serotonin re-uptake, which in the diatom has a key role in growth, reproduction, photoperiodic response and antioxidant defence mechanisms. Moreover, this metabolomic profile also highlighted several promising candidate metabolites and pathways that can be used as fluoxetine exposure biomarkers in diatom cells, namely fatty acid, vitamin, amino acid and pigment metabolites and pathways.

## Data Availability

Data available upon request.
